# HBx-induced S100A9 in NF-κB dependent manner promotes growth and metastasis of hepatocellular carcinoma cells

**DOI:** 10.1038/s41419-018-0512-2

**Published:** 2018-05-24

**Authors:** Liang Duan, Rui Wu, Xiuyu Zhang, Ding Wang, Yan You, Yunyuan Zhang, Lan Zhou, Weixian Chen

**Affiliations:** 1 Department of Laboratory Medicine, The Second Affiliated Hospital of Chongqing Medical University, Chongqing, 400010 China; 2 Department of Laboratory Medicine, The First Affiliated Hospital of Chongqing Medical University, Chongqing, 400016 China; 3 Department of Pathology, The Second Affiliated Hospital of Chongqing Medical University, Chongqing, 400010 China; 4 Department of Laboratory Medicine, The Affiliated Hospital of Qingdao University, Qingdao, 266003 China; 5 Key Laboratory of Diagnostic Medicine Designated by the Chinese Ministry of Education, Chongqing Medical University, Chongqing, 400016 China

## Abstract

Hepatocellular carcinoma (HCC) is associated with hepatitis B virus (HBV) infection. Myeloid-specific S100 proteins (S100s), namely, S100A8, S100A9 and S100A12, have been recently recognized as newly discovered damage-associated molecular patterns (DAMPs) that are correlated with progression in pathogen of infectious diseases. However, whether S100s are regulated by HBV and involved in HBV-related hepatocarcinogenesis are still unclear. Here, we found that all expression levels of myeloid-specific S100s (S100A8, S100A9 and S10012) were elevated in serum and tissue samples from HCC patients. Expression of S100A9 but not S100A8 and S10012 were also higher in blood serum and tissue samples from HBV-positive HCC patients than that in HBV-negative HCC patients. High levels of intracellular and extracellular S100A9 were also confirmed in HepG2 cells expressing 1.3-fold HBV genome or HBV-encoded X protein (HBx) as well as in a stable HBV-producing cell line HepG2.2.15. HBx was shown to facilitate translocation of NF-κB from the cytoplasm to the nucleus, and NF-κB bound to the promoter of S100A9 to enhance its transcription. Silencing S100A9 expression partially blocked HBx-induced growth and metastasis of HepG2 cells both in vitro and in vivo. Further, serum S100A9 levels were found to correlate with TNM stage, extrahepatic metastasis status and HBV DNA load in HBV-related HCC and also had a better diagnostic value for identifying extrahepatic metastasis. Our these data demonstrate that S100A9 plays a pivotal role in HBx-induced growth and metastasis of HCC and may serve as a potential diagnostic marker for extrahepatic metastasis.

## Introduction

Hepatocellular carcinoma (HCC) is the third leading cause of cancer death worldwide and chronic hepatitis B virus (HBV) infection is one of the dominant risk factor for HCC^1,2^. HBV infection contributes to the development, invasion and metastasis of HCC^3,4^. Thus, HBV-related hepatocarcinogenesis is a global health issue. HBV-encoded X protein (HBx), a small 17 kDa soluble protein in the nucleus and cytoplasm of host cells, is known to be essential for HBV-related carcinogenesis^5^. Accumulated data suggest that HBx exerts transcriptional activation by its interaction with nuclear transcription factors (e.g., Oct-1, ATF-2, CREB) and communicates either directly or indirectly with cytoplasmic signal transduction pathway (e.g., PI3-K/Akt, MAK, Ras and Wnt), which accelerate the progress of HCC in many aspects such as inflammation, apoptosis, proliferation, angiogenesis, immune responses and multidrug resistance^6^_._ Although there is extensive evidence to elucidate the implications of HBx in HCC, its precise role in carcinogenic manifestations has not yet been elucidated in detail.

HCC represents one of the most extensively investigated inflammation-related carcinogenesis events since more than 90% of HCCs arise in the context of hepatic injury and inflammation by virus infection^7^. In addition to mediators of inflammation originating externally from HBV infection, some intracellular damage-associated molecular patterns (DAMPs) released by stressed cells undergoing necrosis or secreted by living cells undergoing a life-threatening stress act as endogenous danger signals and gain functions that are distinct from those during normal physiology, modulating inflammatory responses under pathological conditions^8^. DAMPs have been reported to be associated with infection, cellular stress, tissue damage and cancer^9,10^. Some examples of DAMPs include nuclear and cytoplasmic proteins, e.g., high-mobility group box1 (HMGB1), heat shock proteins, myeloid-specific S100 proteins (S100s), histones and interleukin 1 (IL-1) family members.

S100s, namely, S100A8, S100A9 and S100A12, have been recently recognized as newly discovered DAMPs that can activate the innate immune system in response to tissue injury and inflammation due to trauma, infection or cancer^11–14^. S100s (S100A8, S100A9 and S100A12) exert pro-inflammatory properties through their effects on Toll-like receptor-4 (TLR4) and the receptor for advanced glycation end produce (RAGE) signal cascades in inflammatory and immune cells^15,16^, which correlate with many human inflammatory diseases such as rheumatoid arthritis, inflammatory bowel disease and several auto-inflammatory diseases^17,18^. A recent paper also showed that monitoring local S100 levels may be able to sensitively predict disease development^19^. Not just as biomarkers, S100s may actually contribute to the development of some inflammatory diseases in several mouse models, including antigen-induced arthritis, psoriasis and endotoxin-induced septic shock models^20–22^. Of note, S100s have also been over-expressed in many inflammation-associated tumors and contributed to disease progression by activating TLR4 or RAGE signal cascades in tumor cells^23–25^. These observations supported a potential relationship between S100s and inflammation-related cancer.

Recently, S100s have found to be as DAMPs and regulate virus-associated inflammation during virus pathogenesis. For example, S100A9 acts as a DAMP molecular that could enhance inflammation during influenza A virus infection and thereby aggravates pneumonia^26^. S100A8 and S100A9 are also reported to aggravate inflammation in patients with chronic obstructive pulmonary disease infected with rhinovirus (RV)^27^. S100A8 was involved in HPV-induced oral carcinogenesis^28^. However, it is still unclear whether S100s are involved in HBV-related hepatocarcinogenesis.

In the present study, we investigated the expression of S100s (S100A8, S100A9 and S100A12) in HBV-related HCC and explored their roles in HBx-induced growth and metastasis of HCC. We found that S100A9 levels but not S100A8 and S100A12 were significantly elevated in HBV-related HCC tissues and cells. HBx facilitated translocation of nuclear factor (NF)-κB from the cytoplasm to the nucleus and then NF-κB bound to the promoter of S100A9 to enhance its transcription. In addition, by silencing the expression of S100A9, the cell growth and metastasis of HCC cells caused by HBx were significantly impaired in both in vitro and in vivo studies. Moreover, serum S100A9 levels from patients with HBV-related HCC were observed to correlate with the disease progression and also had a better diagnostic value for identifying extrahepatic metastatic status.

## Results

### Expression of S100s in the HBV-negative HCC patients, HBV-positive HCC patients and healthy controls

**Fig. 1:**
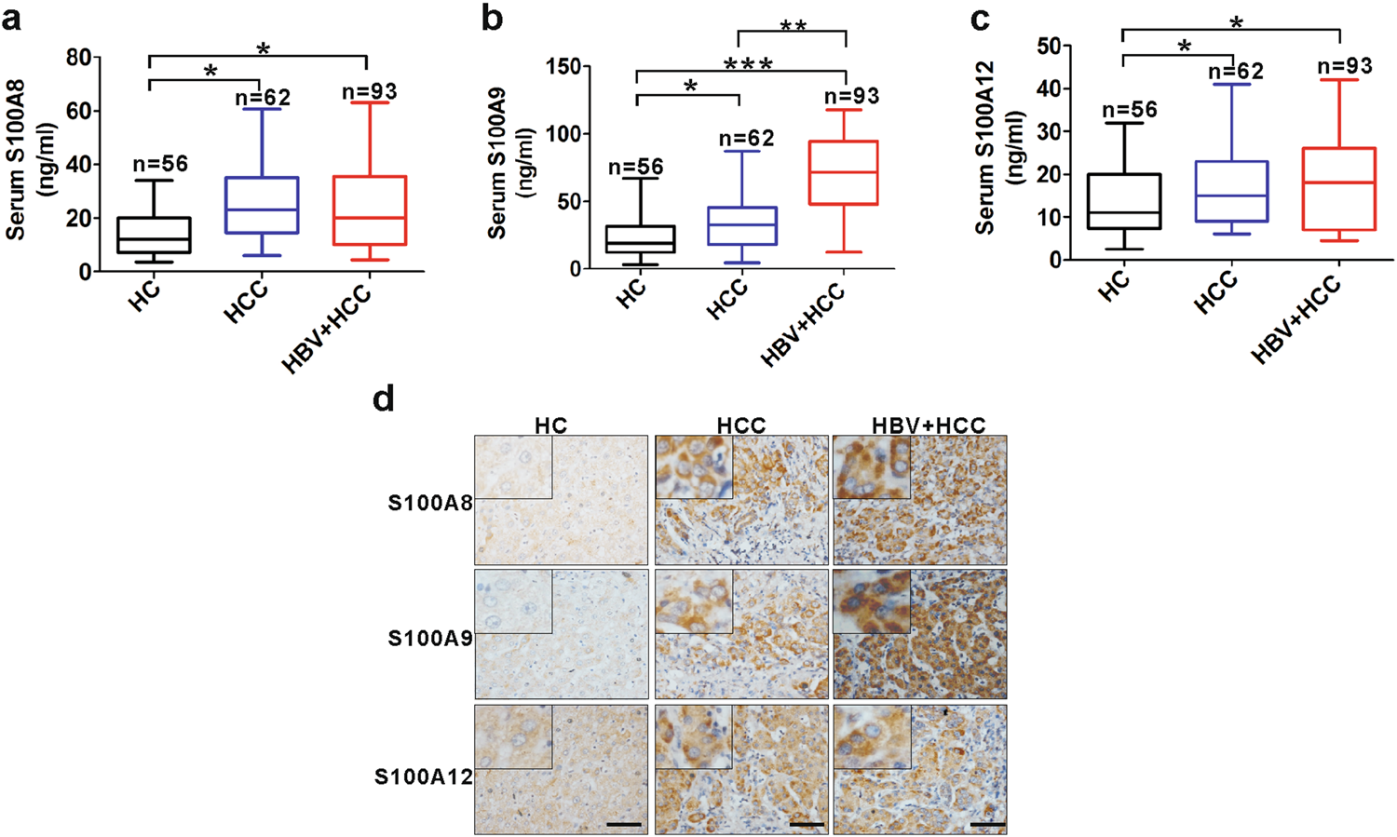
Expression levels of S100A8, S100A9 and S100A12 in healthy controls and patients samples. **a**–**c** ELISA analysis for serum levels of S100A8 (**a**), S100A9 (**b**) and S100A12 (**c**) in healthy controls, HBV-negative HCC patients and HBV-positive HCC patients. **d** Representative IHC staining for S100A8, S100A9 and S100A12 in tissue sections from healthy controls, HBV-negative patients and HBV-positive HCC patients. HC healthy control (*n* = 5), HCC HBV-negative HCC (*n* = 10), HBV+HCC HBV-positive HCC (*n* = 12). Black scale bars = 150 μm; *p < 0.05, **p < 0.01, ***p < 0.001

We analyzed the serum levels of S100A8, S100A9 and S100A12 from HBV-negative HCC patients, HBV-positive HCC patients and healthy controls by enzyme-linked immunosorbent assay (ELISA; Fig. 1a–c). The serum levels of three members were higher from either HBV-negative or -positive HCC patients than that from healthy controls (HCs), while serum levels of S100A9 but not S100A8 and S10012 were higher from HBV-positive HCC patients than that from HBV-negative HCC patients. Further, we also examined the expression of S100A8, S100A9 and S100A12 in sections from HBV-negative and HBV-positive HCC tissues and HCs using immunohistochemical (IHC) staining (Fig. 1d). The results showed that the expression levels of all the three members were significantly higher in tumor cell of intratumoral tissues from HCC patients than from HCs. Of note, the expression of S100A9 was higher in HBV-positive HCC tissues than that in HBV-negative tissues. These histologic results were correspondent to their levels in serum.

### Differential expression of S100s in HepG2 cells transfected with HBV or HBx and in HepG2.2.15 cells

**Fig. 2:**
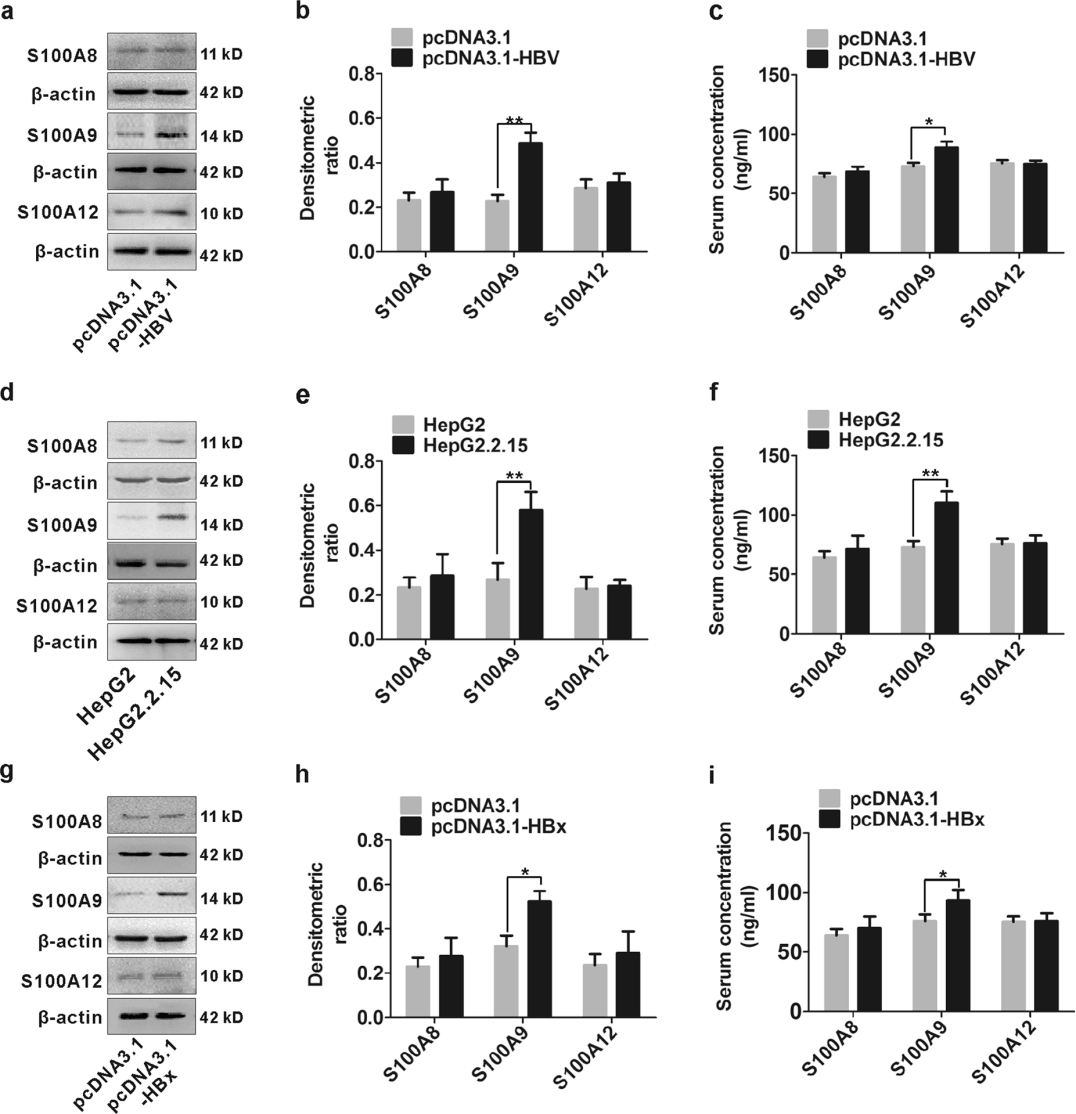
Expression levels of S100A8, S100A9 and S100A12 in HepG2 cells transfected with HBV or HBx and in HepG2.2.15 cells. **a**, **b** Western blot analysis of S100A8, S100A9 and S100A12 in HepG2 cells transfected with HBV expressing plasmid pcDNA3.1-HBV or pcDNA3.1 for 48 h. The relative expression of S100A8, S100A9 and S100A12 is quantified by densitometric ratios and is shown in (**b**). **c** ELISA analysis of S100A8, S100A9 and S100A12 in cultured supernatant of HepG2 cells transfected with pcDNA3.1-HBV or pcDNA3.1 for 48 h. **d**, **e** Western blot analysis of S100A8, S100A9 and S100A12 in HepG2.2.15 cells and its control, HepG2 cell lines. The relative expression of S100A8, S100A9 and S100A12 is quantified by densitometric ratios and is shown in (**e**). **f** ELISA analysis of S100A8, S100A9 and S100A12 in cultured supernatant of HepG2.2.15 cells and its control, HepG2 cell lines. **g**, **h** Western blot analysis of S100A8, S100A9 and S100A12 in HepG2 cells transfected with pcDNA3.1-HBx or pcDNA3.1 for 48 h. The relative expression of S100A8, S100A9 and S100A12 is quantified by densitometric ratios and is shown in (**h**). **i** ELISA analysis of S100A8, S100A9 and S100A12 in cultured supernatant of HepG2 cells transfected with pcDNA3.1-HBx or pcDNA3.1; *p < 0.05, **p < 0.01

To investigate whether S100s expression can be modulated by HBV, we enforced a transient HBV-producing cell line in HepG2 cells by transfection with HBV-expressing plasmid pcDNA3.1-HBV and then S100s were detected by western blot. S100A9 was up-regulated in HBV-transfected HepG2 cells compared to the cells transfected with control pcDNA3.1, but S100A8 and S100A12 were unchanged (Fig. 2a, b). Similar trend was also obtained from their messenger RNA (mRNA) levels (Supplementary Figure 1a). Additionally, S100A9 levels were simultaneously increased in cell supernatant from HBV-transfected HepG2 cells (Fig. 2c). Further, high levels of S100A9 protein were also observed in a stable HBV-producing cell line HepG2.2.15 as well as the cell supernatant compared to the control cell line HepG2, and S100A8 and S100A12 were also unchanged (Fig. 2d–f). Similar trend was also found in their mRNA levels (Supplementary Figure 1b). To determine whether HBx protein participates in this process, we enforced HBx expression in HepG2 cells by transfection with pcDNA3.1-HBx and its control vector pcDNA3.1. S100A9 expression was also up-regulated in HBx-transfected HepG2 cells, and high levels of S100A9 were also observed in cell supernatant from HBx-transfected HepG2 cells, but S100A8 and S100A12 were unchanged (Fig. 2g–i). The change in mRNA levels of S100s was also in correspondence with their protein expression (Supplementary Figure 1c).

### Expression of S100A9 is regulated by HBx-mediated NF-κB activation

**Fig. 3:**
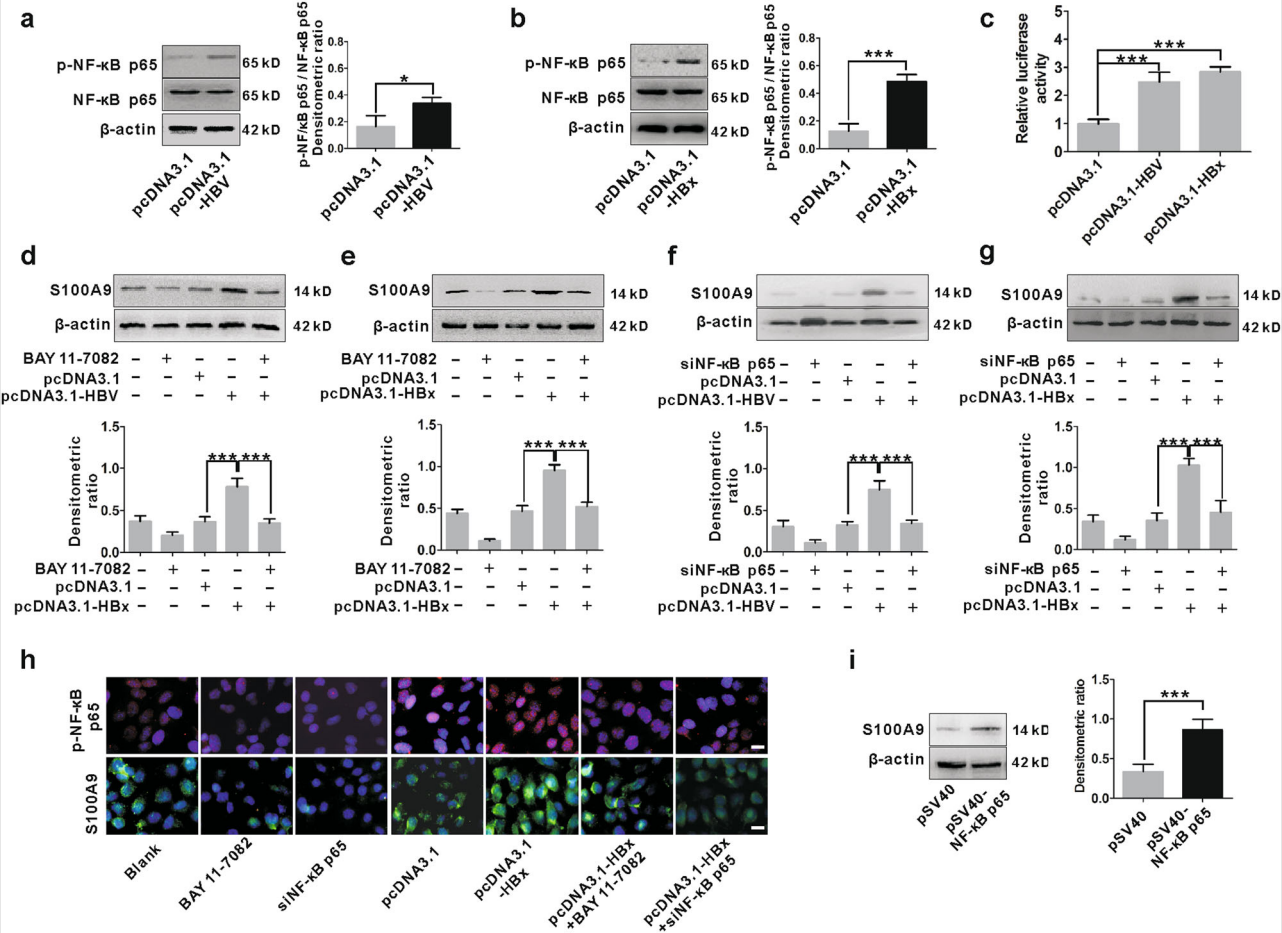
Regulation of S100A9 expression by HBx-mediated NF-κB signaling activation. **a**, **b** Western blot analysis for p-NF-κB p65 levels in HepG2 cells transfected with pcDNA3.1-HBV (**a**) or pcDNA3.1-HBx (**b**) and its control pcDNA3.1 for 48 h. Total NF-κB p65 and β-actin were included as the loading controls. The densitometric ratios were compared to the controls and then normalized to the β-actin and were shown on the right panel, respectively. **c** Dual luciferase reporter assay analysis for transcriptional activity of NF-κB in HepG2 cells co-transfected with pcDNA3.1 or pcDNA3.1-HBV or pcDNA3.1-HBx and luciferase reporter vector (p-Luc-NF-κB). Plasmid RL-TK was also co-transfected to normalize transfection efficiency. Luciferase activity was measured 48 h after transfection. **d**, **e** HepG2 cells were transfected with pcDNA3.1-HBV (**d**) or pcDNA3.1-HBx (**e**) followed by treatment with NF-κB inhibitor BAY 11-7082 (5 μM) for 48 h. The protein levels of S100A9 were analyzed by western blot. The relative expression of S100A9 is quantified by densitometric ratios and is shown below. **f**, **g** HepG2 cells were transfected with pcDNA3.1-HBV (**f**) or pcDNA3.1-HBx (**g**) followed by treatment with siNF-κB p65 for 48 h. The protein levels of S100A9 were analyzed by western blot and quantified by densitometric ratios. **h** Immunofluorescence staining for p-NF-κB p65 and S100A9 in HepG2 cells transfected with pcDNA3.1-HBx followed by treatment with BAY 11-7082 (5 μM) or siNF-κB p65 for 48 h. White scale bars = 10 μm. **i** Western blot analysis of S100A9 in HepG2 cells transfected with pSV40-NF-κB p65 and its control pSV40 for 48 h. β-Actin was included as the loading control. The densitometric ratios were compared to the controls and then normalized to the β-actin and were shown on the right panel; *p < 0.05, ***p < 0.001

HBx is an activator of the transcription factor NF-κB, which is the first HBx-responsive motif to be identified and involved in numerous gene expression^29^. HBx-induced NF-κB activation was confirmed in HepG2 cells transfected by pcDNA3.1-HBV and pcDNA3.1-HBx by western blot and dual luciferase reporter assay, which showed that either HBV or HBx increased the p-NF-κB p65 levels (Fig. 3a, b) and also enhanced transcriptional activity of NF-κB (Fig. 3c). We proceeded to examine whether activation of NF-κB is involved in HBx-induced S100A9 expression. We detected and analyzed S100A9 expression in HepG2 cells transfected with pcDNA3.1-HBV or pcDNA3.1-HBx followed by treatment with or without NF-κB inhibitor BAY 11-7082. NF-κB inhibitor treatment efficiently suppressed the increase of S100A9 levels caused by HBV or HBx (Fig. 3d, e, h). The change in mRNA levels of S100A9 was also in correspondence to its protein levels (Supplementary Figure 2a). Additionally, S100A9 expression was simultaneously analyzed in HepG2 cells transfected with pcDNA3.1-HBV or pcDNA3.1-HBx followed by treatment with or without small interfering RNA (siRNA)-NF-κB p65 (siNF-κB p65). siNF-κB p65 treatment also efficiently suppressed the increase of S100A9 caused by HBV or HBx (Fig. 3f–h). Similar trend was also obtained from its mRNA levels (Supplementary Figure 2b). Further, overexpression of NF-κB p65 by transfection with pSV40-NF-κB p65 also resulted in high levels of S100A9 (Fig. 3i). All these data suggest that S100A9 expression is regulated by HBx-mediated NF-κB activation.

### HBx-mediated NF-κB binds to S100A9 promoter

**Fig. 4:**
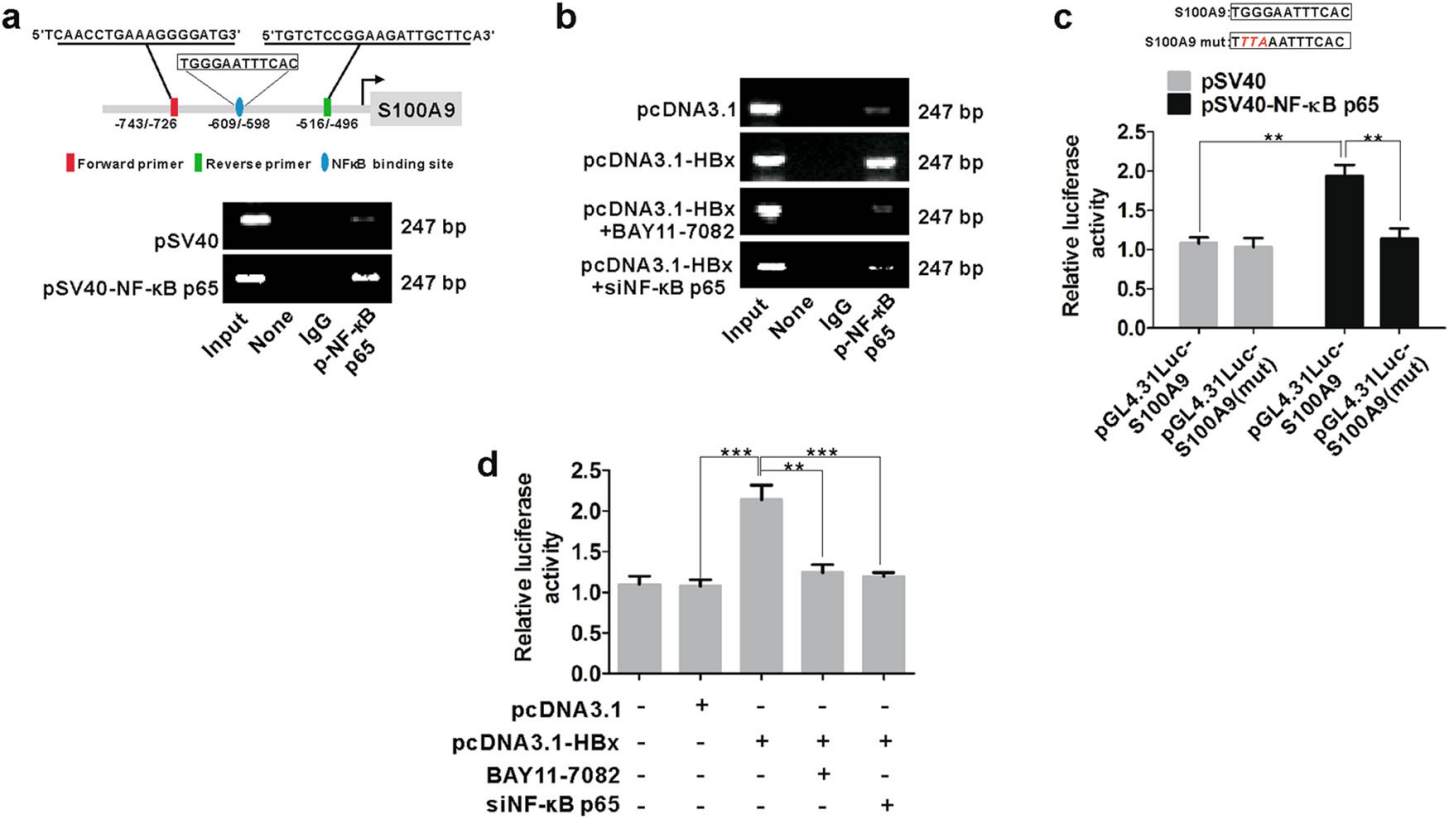
HBx-mediated NF-κB p65 binds to S100A9 promoter. **a** ChIP assay analysis for the interaction between NF-κB p65 and S100A9 promoter in the HepG2 cells transfected with pSV40 or pSV40-NF-κB p65 for 48 h. Input, the sample of DNA from total cell extract. IgG of rabbit was used as the control group. **b** ChIP assay analysis for the interaction between NF-κB p65 and S100A9 promoter in the HepG2 cells transfected with pcDNA3.1 and pcDNA3.1-HBx followed by treatment with BAY 11-7082 (5 μM) or siNF-κB p65 for 48 h. Input, the sample of DNA from total cell extract. IgG of rabbit was used as the control group. **c** Dual luciferase reporter assay analysis for the effect of NF-κB p65 on S100A9 promoter activity in HepG2 cells co-transfected with pSV40 or pSV40-NF-κB p65 and various luciferase reporter vectors (pGL4.31Luc-S100A9 and pGL4.31Luc-S100A9(mut)). Plasmid RL-TK was co-transfected to normalize transfection efficiency. Luciferase activity was measured 48 h after transfection. **d** Dual luciferase reporter assay analysis for the effect of NF-κB p65 on S100A9 promoter activity in HepG2 cells co-transfected with pcDNA3.1 or pcDNA3.1-HBx and luciferase reporter vector (pGL4.31Luc-S100A9) followed by treatment with BAY 11-7082 (5 μM) or siNF-κB p65 for 48 h. Plasmid RL-TK was co-transfected to normalize transfection efficiency; **p < 0.01, ***p < 0.001

To further characterize the mechanism by which HBx-mediated NF-κB activation regulated S100A9 expression, we used chromatin immunoprecipitation (ChIP) to identify the direct binding of NF-κB p65 to the human S100A9 promoter. Fragments of S100A9 promoter were detected in anti-p-NF-κB p65 antibody immunoprecipitated candidates in pSV40-NF-κB p65-transfected HepG2 cells (Fig. 4a), suggesting that NF-κB p65 in the cell nucleus could bind to S100A9 promoter. A very small concentration of fragments was also present in vector-transfected cells (Fig. 4a), which suggested the binding of endogenous p-NF-κB p65 to S100A9 promoter. Additionally, fragments of S100A9 promoter were also detected in HBx-transfected HepG2 cells (Fig. 4b), whereas a very small concentration of fragments was present after NF-κB inhibitor or siNF-κB p65 treatment (Fig. 4b), suggesting that blocking of NF-κB p65 activation efficiently suppressed its binding to S100A9 promoter driven by HBx. S100A9 gene promoter regulated by HBx-mediated NF-κB p65 was further verified by dual luciferase reporter assay, which showed that NF-κB p65 enhanced S100A9 promoter activity in HepG2 cells transfected with pGL4.31Luc-S100A9 promoter (pGL4.31-Luc-S100A9), whereas a complete loss of promoter activity was observed after introduction of specific mutation into the NF-κB p65-binding site of the pGL4.31-Luc-S100A9 (pGL4.31-Luc-S100A9 mut) (Fig. 4c). HBx also enhanced S100A9 promoter activity in HepG2 cells transfected with pGL4.31-Luc-S100A9, but NF-κB inhibitor or siNF-κB p65 treatment resulted in a suppression of S100A9 promoter activity driven by HBx (Fig. 4d). These data suggest that HBx-mediated NF-κB binds to S100A9 promoter and regulated its transcription.

### S100A9 is involved in HBx-mediated growth of HepG2 cells

**Fig. 5:**
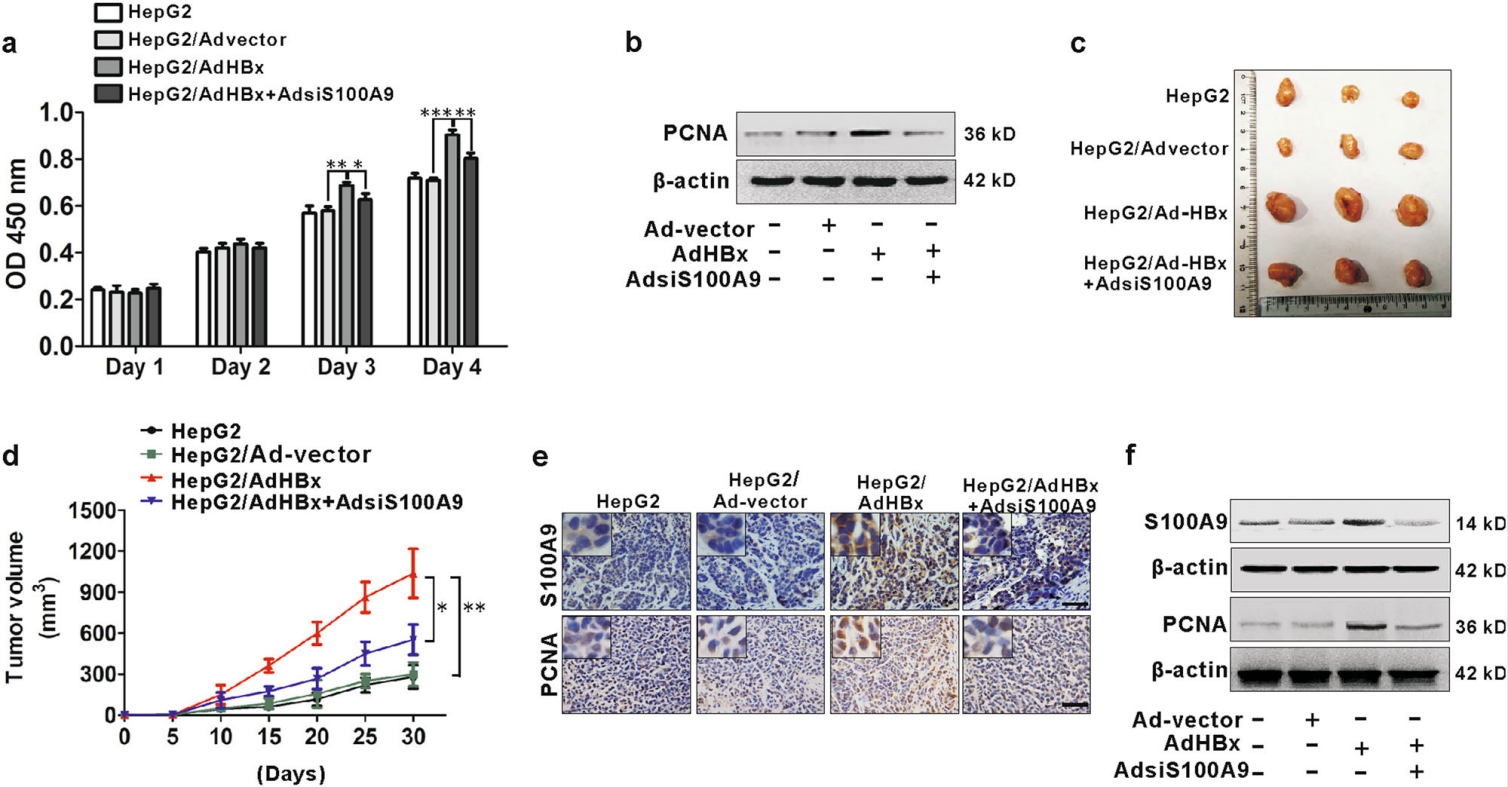
Involvement of S100A9 in HBx-mediated growth of HepG2 cells. **a** CCK8 analysis for HepG2 cells infected with or without Ad-vector, AdHBx, AdHBx and AdsiS100A9 for sequential 4 days. **b** Western blot analysis for PCNA expression in HepG2 cells infected with or without Ad-vector, AdHBx, AdHBx and AdsiS100A9 for 72 h. **c** Images of representative mice bearing tumors derived from mice injected with HepG2 cells infected with or without Ad-vector, AdHBx, AdHBx and AdsiS100A9. **d** Tumor growth curves of all groups. Subcutaneous tumor growth was recorded every 5 days with vernier calipers. **e** IHC staining analysis for S100A9 and PCNA in representative xenograft tumor sections. Black scale bar = 150 μm. **f** Western blot analysis for S100A9 and PCNA expression in representative xenograft tumor tissues; *p < 0.05, **p < 0.01

Cell viability was assayed by CCK-8 assay. HepG2 cells infected with recombinant adenoviruses carrying HBx gene (AdHBx) had an increased cell viability compared to cell infection with control vector (Fig. 5a). It is likely that the increased viable cells by HBx were partially due to promotion of cell growth, because the increased proliferating cell nuclear antigen (PCNA) expression in the enforced HBx expression of cells was confirmed by western blot (Fig. 5b). Previous results showed that S100A9 was up-regulated in HBx-transfected HepG2 cells. We then investigated the role of S100A9 by infection with recombinant adenoviruses carrying S100A9-siRNA gene (AdsiS100A9) for silencing its expression in AdHBx-infected HepG2 cells. We found that silencing of S100A9 inhibited the increase of cell viability and PCNA expression resulted by HBx in HepG2 cells (Fig. 5a, b), suggesting that S100A9 is involved in HBx-mediated growth of HepG2 cells in vitro.

To confirm this effect in vivo, we employed a xenograft nude mice model divided into four groups (HepG2, HepG2/Advector, HepG2/AdHBx and HepG2/AdHBx+AdsiS100A9). Tumor growth was monitored for 30 days after being injected intratumorally with four groups of cells infected by different viral vectors. Compared with Advector group, the tumors derived from the AdHBx group had a much more rapid growth rate (Fig. 5c, d), suggesting that ectopic HBx expression promotes HCC tumor growth in vivo. These were further confirmed by IHC staining or western blot for PCNA expression from various groups, showing that an enhanced immunoreactivity or a high expression of PCNA was observed in AdHBx group (Fig. 5e, f). These results suggest that HBx has a tumor-promoting role in HCC. Also, an increased expression of S100A9 was also detected in AdHBx group compared to the Advector group (Fig. 5e, f). We next analyzed the role of S100A9 in HBx-mediated tumor-promoting effect in HCC. Compared with AdHBx group, the tumors derived from the AdHBx+AdsiS100A9 group had a lower growth rate (Fig. 5c, d). Similarly, decreased expression of S100A9 and PCNA was also observed in AdHBx+AdsiS100A9 group compared to the AdHBx group (Fig. 5e, f). These results suggest that S100A9 is involved in HBx-mediated growth of HCC xenograft tumors in vivo.

### S100A9 is involved in HBx-mediated metastasis of HepG2 cells

**Fig. 6:**
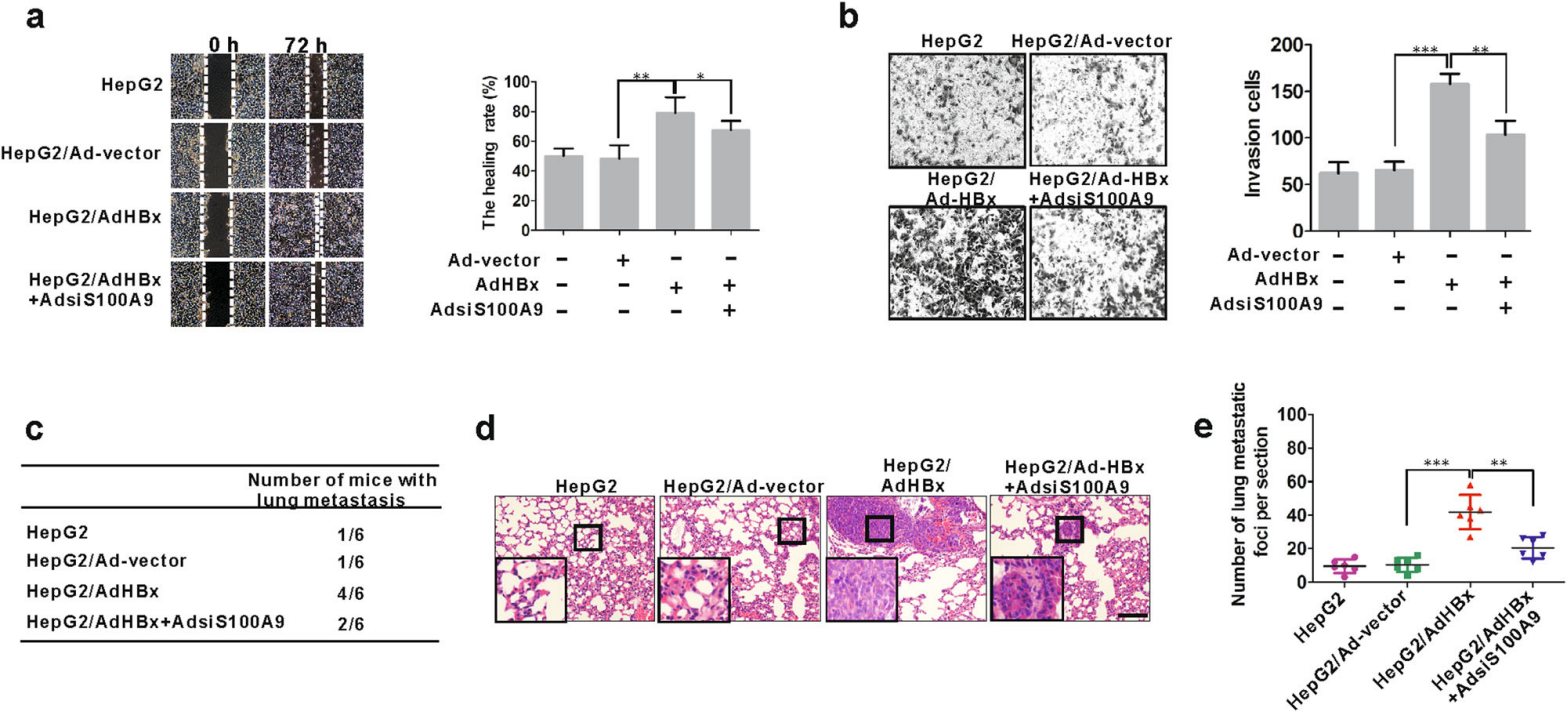
Involvement of S100A9 in HBx-mediated metastasis of HepG2 cells. **a** Wound healing assay for HepG2 cells infected with or without Ad-vector, AdHBx, AdHBx and AdsiS100A9 for 72 h. The incision width of different sites was measured, and average healing rate was calculated and shown in the right panel. **b** Transwell invasion assay for HepG2 cells infected with or without Ad-vector, AdHBx, AdHBx and AdsiS100A9 for 24 h. The representative images of transmembrane cells are shown in the right panel, the mean numbers of transmembrane cells ± SD per microscopic field of three independent experiments are quantified in the right panel. Magnification, ×150. **c** The total numbers of mice with distant lung metastasis after injection of HepG2, HepG2/Ad-vector, HepG2/AdHBx, HepG2/AdHBx+AdsiS100A9 cells for 5 weeks. **d**, **e** The number of metastatic foci per section in lungs from individual mouse with injection of HepG2 cells infected with or without Ad-vector, AdHBx, AdHBx and AdsiS100A9. Black scale bars = 200 μm; *p < 0.05, **p < 0.01, ***p < 0.001

Cell migration and invasion play crucial roles in the process of tumor metastasis. Wound healing assay and transwell assay were used to evaluate in vitro cell migration and invasiveness, respectively. After infection with AdHBx in HepG2 cells for 72 h, wound closure rate was increased by 63.8% compared to that in the cell infection with control vector (Fig. 6a). Similarly, after infection with AdHBx in HepG2 cells for 24 h, the number of transmembrane cells was increased by 140% compared to that in the cell infection with control vector (Fig. 6b). These results suggest that HBx promotes migration and invasion of HepG2 cells in vitro. We then elucidate the role of S100A9 by infection with AdsiS100A9 for silencing its expression in AdHBx-infected HepG2 cells. We found that silencing of S100A9 partially inhibited the increase of cell migration and invasion mediated by HBx in HepG2 cells in vitro (Fig. 6a, b). These results suggest that S100A9 is involved in HBx-mediated migration and invasion of HepG2 cells in vitro.

To examine the effect of S100A9 on HBx-mediated metastatic property in vivo, we employed the HepG2 cells with various treatments (HepG2, HepG2/Advector, HepG2/AdHBx, HepG2/AdHBx+AdsiS100A9) as the cell model for in vivo metastasis studies. At 5 weeks after the injection by tail vein of nude mice, the metastasis foci were examined in the lungs of mice. The pulmonary metastases were first examined through organ anatomy and then pathologic examination. HBx not only significantly increased the number of mice with distant pulmonary metastasis (Fig. 6c), but also dramatically increased the number of metastatic tumors in the lung of each mouse (Fig. 6d, e). Strikingly, the group of mice receiving AdsiS100A9 treatment for silencing S100A9 expression showed significantly fewer metastatic colonies in the lung (Fig. 6d, e). These results indicated that S100A9 is involved in HBx-mediated metastatic property in vivo.

### Diagnostic significance of serum S100A9 levels in patients with HBV-positive HCC

**Fig. 7:**
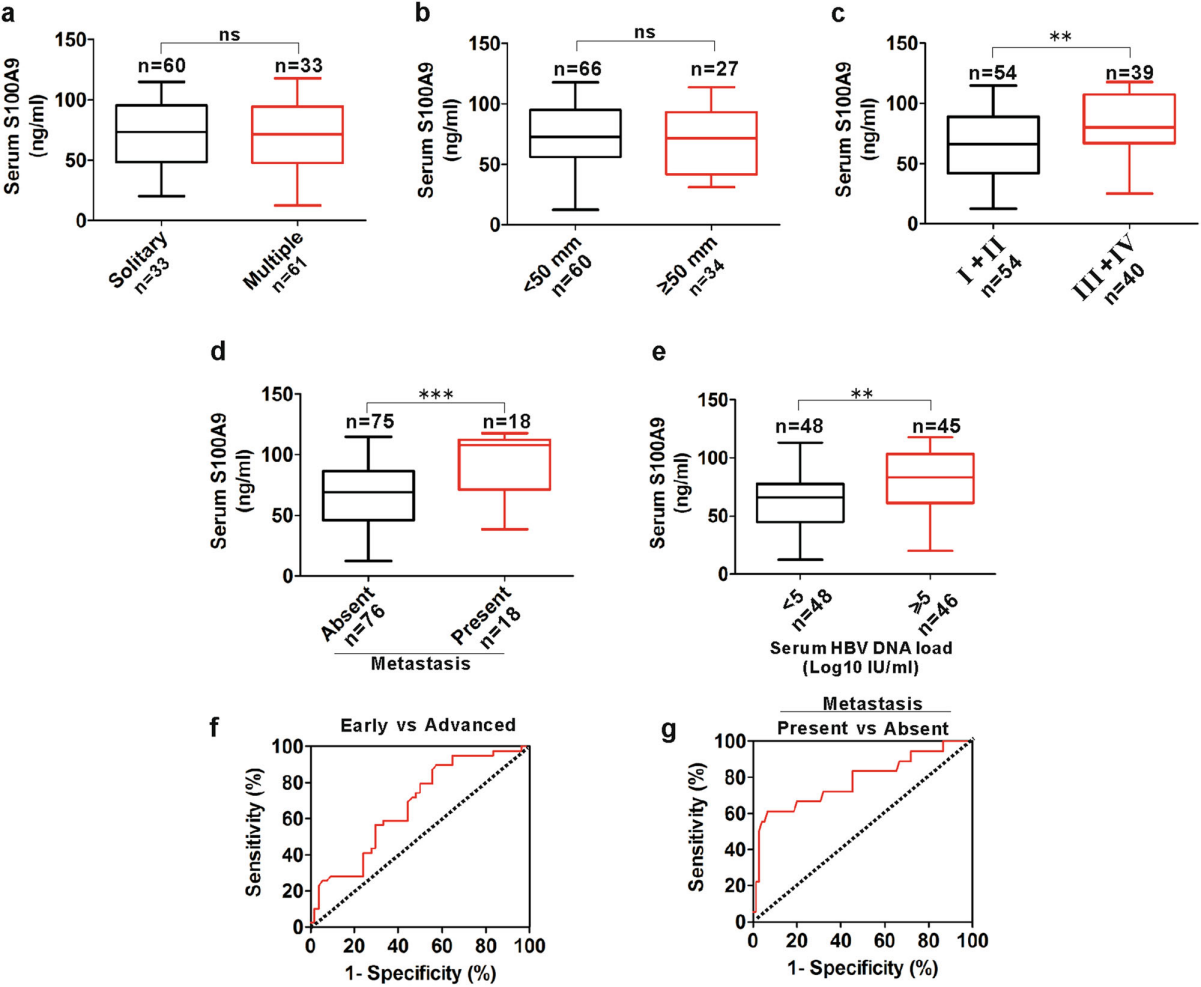
Correlation of serum S100A9 levels with clinical-pathological parameters and its diagnostic value for HBV-positive HCC. **a** Distribution of serum S100A9 levels in patients with various tumor numbers (solitary vs.multiple). **b** Distribution of serum S100A9 levels in patients with various tumor diameters (<50 mm vs .≥50 mm). **c** Distribution of serum S100A9 levels in patients with early and advanced stages (I/II vs. III/IV). **d** Distribution of serum S100A9 levels in patients with and without extrahepatic metastasis. **e** Distribution of serum S100A9 levels in patients with low and high serum HBV load. **f** ROC curve of serum S100A9 for detecting with advanced stages from early stages in HBV-positive HCC. **g** ROC curve of serum S100A9 for detecting extrahepatic metastasis from none in HBV-positive HCC; **p < 0.01, ***p < 0.001; ns no significance

Based on the elevated S100A9 levels and its promotional role on carcinogenesis and metastasis, we next evaluated the correlation of serum S100A9 levels and clinical-pathological parameters such as tumor number, tumor diameter, TNM (tumor, node, metastasis) stage, extrahepatic metastasis status and HBV DNA load. There were no obvious differences in samples with different tumor numbers (solitary/multiple) (Fig. 7a) and tumor diameters (<50 mm/ ≥ 50 mm) (Fig. 7b). Advanced HCC serum samples (III/IV) had higher S100A9 levels than early-stage HCC samples (I/II) (Fig. 7c). Extrahepatic metastasis samples had higher S100A9 levels than without extrahepatic metastasis samples (Fig. 7d). High HBV load samples had higher S100A9 levels than low HBV DNA load (Fig. 7e). These findings indicate that serum S100A9 levels are correlated with TNM stage, extrahepatic metastasis status and HBV DNA load in HBV-related HCC.

We next evaluated whether serum S100A9 can be regarded as a biomarker of HCC progression. Receiver operating characteristic (ROC) analysis showed that serum S100A9 levels had weaker diagnostic value for identifying advanced stage from early stage, which yielded an area under the ROC curve (AUC) of 0.67.4 (95% confidence interval (CI), 0.535 to 0.725) with 89.7% sensitivity, 42.5% specificity and 66.1% accuracy (Fig. 7f). Strikingly, serum S100A9 levels had better diagnostic value for identifying extrahepatic metastasis, which yielded an AUC of 0.781 (95% CI, 0.646 to 0.917) with 62.3% sensitivity, 93.3% specificity and 77.8% accuracy (Fig. 7g). These findings indicate that the identified serum S100A9 may efficiently predict extrahepatic metastasis.

## Discussion

Involvement of DAMPs is being uncovered in almost all aspects of cancer biology, such as proliferation, tumorigenesis, apoptosis, invasion, metastasis and angiogenesis^13^. Given that S100s have been reported to be as DAMPs and involved in virus-associated pathogenesis, we wonder if S100s are involved in HBV-related hepatocarcinogenesis. Our data demonstrated that enhanced transcriptional activity of NF-κB caused by HBx resulted in elevated S100A9 expression, which subsequently contributed to the growth and metastasis of HCC cells. Additionally, monitoring serum S100A9 levels may be able to predict disease development in a more sensitive and reliable way.

S100s are a family of more than 20 homologous intracellular proteins characterized by calcium-binding EF hand motifs, low molecular weights, ability to form homodimers and heterodimers and oligomers, and tissue-specific expression. Extracellular S100s secreted from cells function as DAMP ligands for cell surface receptors, activating signaling cascades and triggering cellular response. The most well-studied extracellular effects relate to the myeloid-specific S100s (S100A8, S100A9 and S100A12), which have been recently recognized as the newly discovered DAMPs^11^. During some infectious diseases, serum and/or local levels of S100s can be up-regulated and their levels may be related to the disease progression. For example, S100A8, S100A9 and S100A12 are highly expressed in *Helicobacter pylori*-infected gastric mucosa^30^. Serum S100A8 and S100A9 levels reflect disease activity in ANCA-associated vasculitis, glomerulonephritis and systemic lupus erythematosus^31,32^. S100A12 concentrations are significantly increased in acute otitis media infected with *Streptococcus pneumoniae*, *nontypeable Haemophilus influenzae*^33^. Our present study also showed that patients with HBV-positive HCC had higher serum S100A9 levels, which also correlated with disease progression and may efficiently predict extrahepatic metastasis. However, pending additional work on larger cohorts of patients and usage of serum S100A9 in diagnosis of other virus-associated cancer are promising.

S100A9 is a member of the S100 family which is characterized by the presence of two Ca^2+^ binding sites of the EF-hand type. It has been demonstrated that S100A9 was correlated with the extent of inflammation and may even actually contribute to the development in some inflammatory diseases including cancers^23^. Our recent study as well as other studies also pointed out an elevated S100A9 expression in HCC^34,35^. However, the mechanism for S100A9 up-regulation has not been clarified. Given that most HCC patients are HBV positive, we hypothesized that S100A9 up-regulation may be related to HBV infection. It is well known that HBx plays an important role in viral pathogenesis and hepatocarcinogenesis due to its interaction with various signal transduction pathways and regulation of various functional genes^6^. Here, we revealed that HBx protein enhanced S100A9 expression. Previous reports have shown that HBx protein activates NF-κB by facilitating translocation of NF-κB from the cytoplasm into the nucleus^36^. Therefore, we hypothesized that activation of NF-κB by the HBx may induce S100A9 transcription. Our data indicated that the promoter of S100A9 was activated by HBx-mediated NF-κB activation. Thus, we conclude that the HBx protein promotes S100A9 expression in a NF-κB-dependent manner.

High levels of S100A9 were also observed in cell supernatant from HBV or HBx-treated HepG2 cells. We speculate that these proteins may be released from high levels of intracellular S100A9 caused by HBV or HBx. With respect to its secretion, several investigators found that cytosolic S100A9 lack leader sequences and cannot be exported via the classical Golgi pathway and they tend to believe that these proteins are passively released from dead cells during the disease development^37^. On the contrary, other investigators have suggested that these proteins can be secreted out in an energy-dependent, tubulin-dependent, protein kinase C-related alternative pathway^38,39^. Therefore, there is still need to investigate the secretary mechanism of S100s in further studies.

One of the most well studies of S100A9 protein is working as DAMP molecule in tumor microenvironment in inflammation-associated cancer that can active several pattern recognition receptors, including RAGE and TLR4, activating signaling cascades and thereby facilitating carcinogenesis and tumor progression. It has been reported that in the microenvironment of cancer, S100A9 binds to RAGE on myeloid-derived suppressor cells (MDSCs) and promotes MDSC migration to the tumor site and represses host-mediated antitumor immune response against cancer cells, thereby facilitating carcinogenesis and tumor progression^40,41^. S100A9 also interacts with TLR4 and promotes tumor growth in prostate tumor and lymphoma models^42^. Our previous studies demonstrate that extracellular S100A9 directly binds to RAGE on HCC cells and stimulates RAGE-dependent MAPK signaling cascades, promoting cell growth and invasion in HCC in vitro^34,43^. Our present study indicated that HBx promoted HCC cell growth and metastasis both in vitro and in vivo, which was partially mediated by enhanced expression of S100A9 from liver cancer cell expressing HBx. Silencing of S100A9 expression by AdsiS100A9 significantly reduced the growth and invasion of HCC cells both in vitro and in vivo. Therefore, S100A9 may be a viral oncogene during HBV-related hepatocarcinogenesis.

A unique characteristic of S100s is that some members are actively released from cells into the extracellular space and even located in serum where they are correlated with disease progression. Recently, a growing body of evidence points to the elevated serum levels of S100A9 in few types of inflammation-associated cancers, including prostate cancer^44^ and colorectal cancer^45^. As a classical inflammation-associated cancer for HBV-related HCC, the elevated levels of serum S100A9 were also observed, and its serum levels had a better diagnostic value for identifying extrahepatic metastasis. All these evidences suggest that S100A9 might be the future candidate marker for diagnosis of inflammation-associated cancer. Therefore, pending additional work on larger samples of various types of inflammation-associated cancers and usage of serum levels of S100A9 for diagnosis is promising.

Taken together, these data demonstrate that S100A9 plays a pivotal role in HBx-induced HCC growth and metastasis and may serve as a potential diagnostic marker for extrahepatic metastasis of HCC. Considering the role of HBV in the development and progression of HCC, our data support the notion that S100A9 may serve as a potential target for the development of novel anticancer agents against HBV-related HCC. Future studies dealing with identification and characterization of host-derived molecular patterns (e.g. DAMPs) during virus infection may lead to the development of measures to combat infection-associated inflammatory cancer.

## Materials and methods

### Patients

For the tissue samples, 12 clinical HBV-positive and 10 HBV-negative HCC samples were collected from patients who had undergone HCC resection at the Second Affiliated Hospital of Chongqing Medical University. Simultaneously, five age- and sex-matched distal normal tissues were collected from HCC patients who had undergone HCC resection and were recruited in this study, and these healthy controls (HCs) were subjected to blood test to rule out the HBV infection. In all, 93 HBV-positive and 62 HBV-negative HCC serum samples were also collected from patients recruited from the Second Affiliated Hospital of Chongqing Medical University along with 49 age- and sex-matched HCs, and these HCs were subjected to blood test to rule out the HBV infection. The patients received no chemotherapy, or radiotherapy before surgery, and the written informed consent was received from all participants. This study was approved by the Ethics Committee of the Second Affiliated Hospital of Chongqing Medical University. The characteristics of the enrolled individuals are in Table 1.

**Table 1:** The characteristics of enrolled individuals

	HCC		HCs (*n* = 49)
Parameter	HBV-negative (*n* = 62)	HBV-positive (*n* = 93)	
Gender (male, %)			
Mail	37	58	29
Female	25	35	20
Age (years)			
<60	28	61	30
≥60	34	32	19
TNM stage			
I + II	38	54	N/A
III + IV	24	39	N/A
Tumor diameter			
<50 mm	40	66	N/A
≥50 mm	22	27	N/A
Tumor number			
Solitary	30	33	N/A
Multiple	32	60	N/A
HBV DNA (log_10_ IU/ml)			
<5	N/A	48	N/A
≥5	N/A	45	N/A
Extrahepatic metastasis status			
Absent	37	59	N/A
Present	25	18	N/A

*N/A* not available

### Cell culture

HepG2 cells (American Type Culture Collection, USA) and HepG2.2.15 cells (Chongqing Medical University) that constitutively replicated HBV were cultured in Dulbecco’s modified Eagle's medium with 10% fetal bovine serum (FBS, Hyclone, USA). Cells were incubated at 37 °C in a humidified atmosphere with 5 % CO_2_.

### Plasmids and recombinant adenoviruses

The expression vector of pcDNA3.1-HBx was constructed by inserting HBx DNA fragments into pcDNA3.1 vector. The 1.3-fold HBV genome fragment was amplified from pGEM-HBV1.3, and the amplified 1.3-fold HBV genome fragments was cloned into pcDNA3.1 plasmid for the construction of pcDNA3.1-HBV plasmid. These plasmids were gifted by Professor Ailong Huang (Institute for Viral Hepatitis, Chongqing Medical University). The recombinant adenoviruses carrying human S100A9 gene (AdS100A9) and S100A9-siRNA gene (AdsiS100A9) and their control vectors were constructed in our laboratory. The recombinant adenoviruses carrying HBx gene and its control vector were kindly presented by Professor Tao Feng (Molecular Medicine and Cancer Research Center, Chongqing Medical University)^46^. All these recombinant adenoviruses were amplified in HEK293 cells before use. The expression vector of pSV40-NF-κB p65 and its control vector pSV40 were purchased from GeneCopoeia. Luciferase report vectors pGL4.31-Luc-S100A9 and pGL4.31-Luc-S100A9 mut were constructed by inserting S100A9 promoter and mutational S100A9 promoter fragments into pGL4.31-Luc vector. Luciferase report vector p-Luc-NF-κB and its control vector were used before and were gifted by Professor T.C. He (The University of Chicago Medical Center, Chicago, IL, USA)^47^.

### Reagents and antibodies

The primary antibodies used for this study were as follows: the mouse anti-S100A8 monoclonal antibody (Cat. no. 48352, Santa Cruz, USA), mouse anti-S100A9 monoclonal antibody (Cat. no. 58706, Santa Cruz, USA) and mouse anti-S100A12 monoclonal antibody (Cat. no. 101347, Santa Cruz, USA), rabbit anti-proliferating cell nuclear antigen (PCNA) polyclonal antibody (Cat. no. 18197, Abcam, UK), rabbit anti-phospho-NF-κB p65 (p-NF-κB p65) monoclonal antibody (Cat. no. 3033, CST, USA), rabbit anti-NF-κB p65 monoclonal antibody (Cat. no. 8242, CST, USA), mouse anti-β-actin monoclonal antibody (Cat. no. 47778, Santa Cruz, USA). The NF-κB inhibitor BAY 11-7821 was purchased from Beyotime. siNF-κB p65 (sense, 5′-CCAUCAACUAUGAUGAGUUdTdT-3′ and antisense, 3′-dGdTGGUAGUUGAUACUACUCAA-5′) was designed to target the NF-κB p65 and was produced by GenePharma^48^. The cells were transfected with siRNA using Lipofectamine™ 2000 (Invitrogen) according to the manufacturer’s instructions.

### IHC staining

For IHC analysis, the sections from the formalin fixed, paraffin-embedded tissues were deparaffinized and rehydrated. Then, the sections were boiled for 10 min in 0.01 M citrate buffer and incubated with 0.3% H_2_O_2_ in methanol to block endogenous peroxidase. The sections were incubated with the anti-S100A8, anti-S100A9, anti-S100A12 or anti-PCNA antibody (1:200 dilution, respectively), followed by incubation with secondary antibody tagged with the peroxidase enzyme and were visualized with 0.05% DAB (3,3'-diaminobenzidine) until the desired brown reaction product was obtained. All slides were observed under a Nikon E400 Light Microscope and representative photographs were taken.

### Immunofluoresence staining

The cells were plated and cultured onto cleaned-up cover slips, and were washed with phosphate-buffered saline (PBS) and fixed in 4% paraformaldehyde, then permeabilized with 0.2% Triton X-100. Cover slips were rinsed and incubated with blocking serum for 15 min at 37 ℃ and then incubated with anti-p-NF-κB p65 or anti-S100A9 antibody (1:100 dilution, respectively) overnight at 4 ℃. After three washes with PBS, the cells were stained with the corresponding Alexa Fluor 647-conjugated or fluorescein isothiocyanate-conjugated anti-secondary antibodies. To visualize nuclei, cells were stained with 10 µg/ml DAPI (4',6-diamidino-2-phenylindole). The fluorescent images were then observed and analyzed using a multilaser confocal microscope.

### ELISA

Serum samples collected were first blinded and then tested in duplicate. S100A8, S100A9 and S100A12 levels in serum samples as well as S100A12 levels in culture supernatants were measured using human S100A8 (JYM0540Hu, JYM, China), S100A9 (JYM0539Hu, JYM, China) and S100A12 (JYM1696Hu; JYM, China) ELISA kits according to the manufacturer’s recommended procedure.

### Real-time quantitative PCR analysis

HepG2 cells were transfected with pcDNA3.1-HBV or pcDNA3.1-HBx followed by treatment with and without BAY 11-7082 (5 μM) or siNF-κB p65 for 24 h and then lysed with Trizol (Invitrogen, Carlsbad, CA, USA). Complementary single-stranded DNA was synthesized from total RNA by reverse transcription (TaKaRa, Japan). Primers were also synthesized by Invitrogen. PCR primers were as follows: S100A8 primers: (forward) 5′-TGTCTCTTGTCAGCTGTCTTTCA-3′ and (reverse) 5′-CCTGTAGACGGCATGGAAAT-3′; S100A9 primers: (forward) 5′-GGAATTCAAAGAGCTGGTGC-3′ and (reverse) 5′-TCAGCATGATGAACTCCTCG-3′; S100A12 primers: (forward) 5′-CACATTCCTGTGCATTGAGG-3′ and (reverse) 5′-TGCAAGCTCCTTTGTAAGCA-3′; GAPDH primers (forward) 5′-CAGCGACACCCACTCCTC-3′ and (reverse) 5′-TGAGGTCCACCACCCTGT-3′. Reactions were performed in triplicate using SYBR Green master mix (TaKaRa, Japan) and normalized to GAPDH mRNA level using the ΔΔCt method.

### Cell proliferation assay

Untreated, Ad-vector-treated, AdHBx-treated and AdHBx+AdsiS100A9-treated HepG2 cells were seeded at a density of 2 × 10^3^ cells per well in a 96-well plate for different time intervals as indicated, and cell proliferation was determined by CCK-8 assay using a Cell Counting Kit (Dojindo, Japan) following the manufacturer’s protocol. The final absorbance was measured daily for the following 4 days at 450 nm using a microplate reader. Each condition was done in quintuplicate, and the experiment was repeated thrice.

### Cell migration assay

Cell migration ability was analyzed by means of wound scratch assay. Untreated, Ad-vector-treated, AdHBx-treated and AdHBx+AdsiS100A9-treated HepG2 cells were collected and seeded in 6-well plates. After the cells were attached to the wall, wound was created at the center of the culture and the cells were washed with serum-free medium, cultured with 1% FBS. Images were taken under a microscope immediately after the incision was made. The incision width of the different sites was measured, and the average wound closure rate was calculated. The wound closure rate was calculated as: (0 h incision width − 72 h incision width)/0 h incision width × 100%.

### Transwell invasion assay

The chamber of non-type I-collagen-coated 24-well culture insert (MILLIPORE, USA) was used, and the upper side of the insert was coated with ECM gel (SIGMA, USA). Briefly, cells were placed in the upper chamber (2 × 10^5^ cells) and incubated with various treatments in serum-free medium, while there was only the medium (600 μl/each insert) with 20% FBS in the lower chamber. After incubation for 24 h, the transmembrane cells were dried, fixed with methanol, stained with 0.1% crystal violet and counted under microscopy at 150×. The experiment was performed thrice.

### In vivo tumor growth and metastasis

The in vivo tumor growth were performed as previously described^49^. Briefly, 6–8-week old female nude mice were randomly divided into four groups (*n* = 3 for each group). Untreated, Ad-vector-treated, AdHBx-treated and AdHBx+AdsiS100A9-treated HepG2 cells (1 × 10^7^/each nude mouse) were suspended in 200 µl PBS, and then were injected subcutaneously into the posterior flank position of nude mice. Subcutaneous tumor growth was recorded every 5 days with vernier calipers. Tumor volume was calculated using the formula: π/6 × (*R*max x *R*min^2^), where *R* is the tumor diameter. The mice were killed after 30 days, and the tumor tissues were collected, fixed in buffered formaldehyde, embedded in paraffin and sectioned for further histological and immunohistochemical analysis. For the in vivo metastasis assays, cells were resuspended in PBS at a concentration of 5 × 10^7^ cells/ml. Cell suspension (0.1 ml) was injected into tail veins of nude mice (*n* = 6 for each group). All of the mice were killed by CO_2_ 35 days after inoculation. The livers and lungs were dissected from mice and fixed in 4 % paraformaldehyde in PBS overnight and subsequently embedded in paraffin wax. Sections were cut and stained with hematoxylin and eosin for histological analysis. All the experimental procedures were conducted in accordance with the guidelines established by the University Animal Care and Use Committee for Laboratory Animal Research.

### Western blot assay

Western blot analysis was applied to evaluate levels of S100A8, S100A9, S100A12, p-NF-κB p65, NF-κB p65 and PCNA in tissues or cells. Briefly, the tissues and cells were collected and washed with ice-cold PBS, then lysed on ice in radio immunoprecipitation assay buffer. Samples containing equal amount of proteins were separated in 10% sodium dodecyl sulfate–polyacrylamide gel electrophoresis and blotted onto the polyvinylidene difluoride membranes. Then, the membranes were blocked with 5% bovine serum albumin and incubated with anti-S100A8, anti-S100A9, anti-S100A12, anti-p-NF-κB p65, anti anti-NF-κB p65, anti anti-PCNA or anti-β-actin antibody (1:1000 dilution, respectively), followed by incubation with secondary antibodies conjugated with horseradish peroxidase. The proteins of interest were detected using the SuperSignal West Pico Chemiluminescent Substrate kit. The results were recorded by the Bio-Rad Electrophoresis Documentation (Gel Doc 1000, Bio-Rad, USA) and Quantity One Version 4.5.0.

### ChIP assay

ChIP was performed as described previously^50^. Briefly, cells were fixed with 1% formaldehyde. Immunoprecipitation was performed with anti-p-NF-κB p65 or normal control IgG antibody coupled to protein A/G-Sepharose. Immunoprecipitated chromatin-derived DNA was analyzed by PCR with primers specific for the S100A9 promoter DNA at the p-NF-κB p65 binding region (positions from −496 to −743) compared to the input mixture. The primers used were as follows: forward primer 5′-TCAACCTGAAAGGGGATG-3′ and reverse primer: 5′-TGTCTCCGGAAGATTGCTTCA-3′. PCR products were resolved on agarose gel and visualized with ethidium bromide.

### Luciferase assay

Luciferase activity in cell lysates was detected with a dual luciferase reporter assay system (Promega; USA). Cells were collected after co-transfecting luciferase report vector (p-Luc-NF-κB or pGL4.31Luc-S100A9 or pGL4.31Luc-S100A9(mut)) with either pcDNA3.1-HBx or pSV40-NF-κB p65 followed by treatment with and without BAY 11-7082 (5 μM) or siNF-κB p65 for 48 h, and luciferase activity was detected in the lysates using a GloMax microplate luminometer (Promega, USA). Luciferase activity was normalized by co-transfecting with pRL-TK (Promega).

### Statistical analysis

The differences in the results of tissues and cells were analyzed using one-way analysis of variance followed by the Student–Newman–Keuls test, and the differences in the results of serum S100A8, S100A9 or S100A9 levels were performed using Mann–Whitney test. ROC curves were generated to classify patients in different groups, as well as for the evaluation of the diagnostic potential of serum S100A9 via calculation of the area under the ROC curve, sensitivity and specificity according to standard formulas. All statistical analyses were performed using GraphPad Prism software (GraphPad Software, CA, USA). Statistical differences are presented at probability levels of *p* < 0.05, *p* < 0.01 and *p* < 0.001.

## Electronic supplementary material

Supplementary figures
