# Supplementary material for: HBx-induced S100A9 in NF-κB dependent manner promotes growth and metastasis of hepatocellular carcinoma cells
Source: Cell Death Dis. 2018 May 24;9(6):629. doi: 10.1038/s41419-018-0512-2 (PMC5967311; doi:10.1038/s41419-018-0512-2)
Supplement: Supplementary file 1 — Supplementary figures [file 41419_2018_512_MOESM1_ESM.doc]

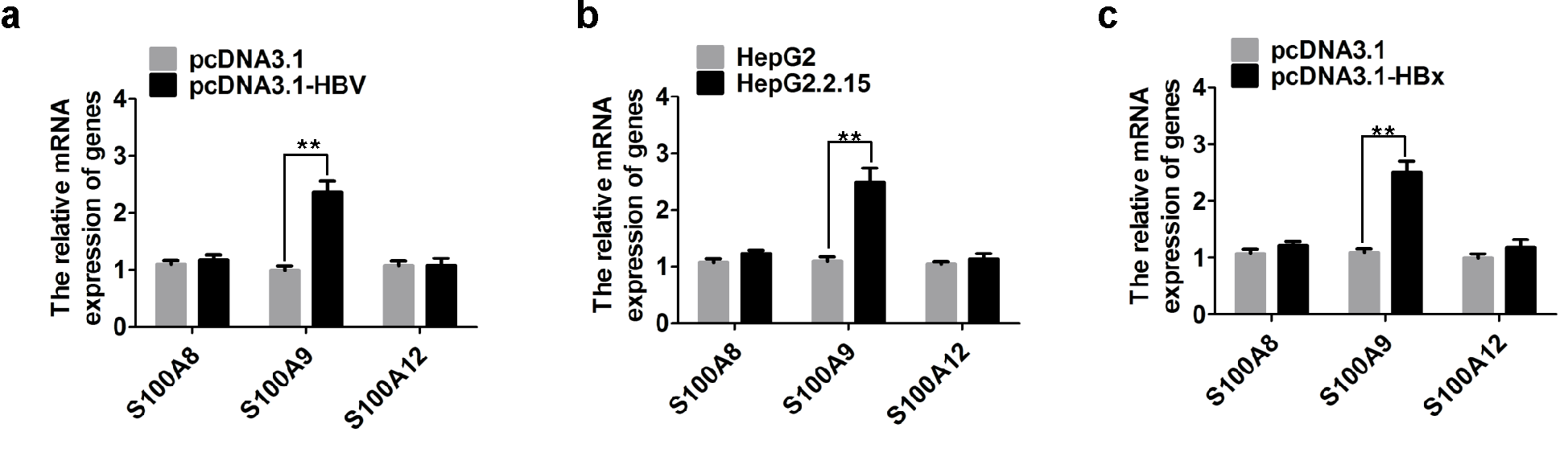
Supplementary figure 1 Gene expression levels of S100A8, S100A9, and S100A12 in HepG2 cells transfected with HBV or HBx and in HepG2.2.15 cells. (a) Real-time PCR analysis for mRNA levels of S100A8, S100A9, and S100A12 in HepG2 cells transfected with HBV expressing plasmid pcDNA3.1-HBV or pcDNA3.1 for 24 h. (b) Real-time PCR analysis for mRNA levels of S100A8, S100A9, and S100A12 in HepG2.2.15 cells and its control, HepG2 cell lines. (c) Real-time PCR analysis for mRNA levels of S100A8, S100A9, and S100A12 in HepG2 cells transfected with pcDNA3.1-HBx or pcDNA3.1 for 24 h. **p<0.01.


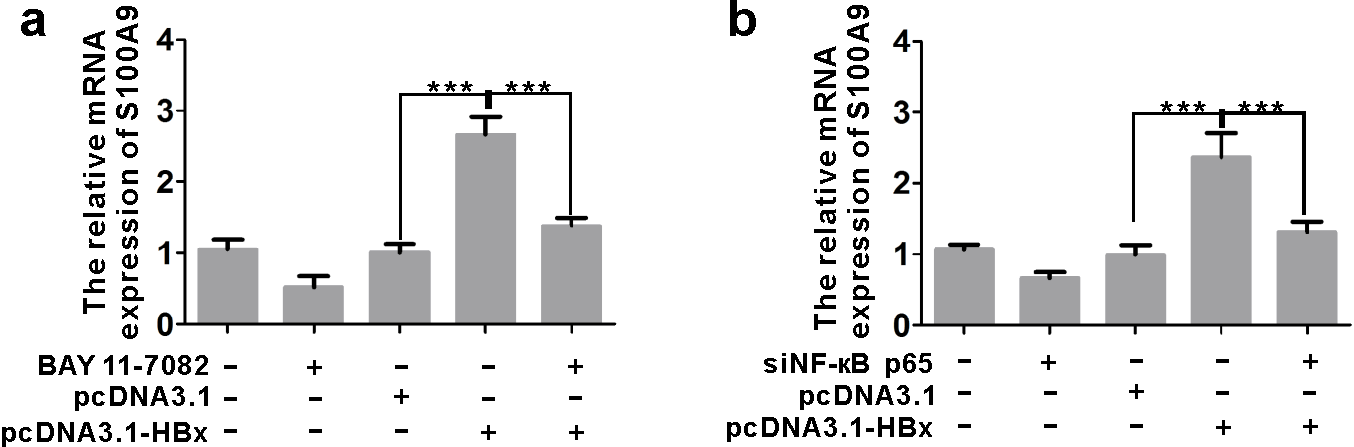


Supplementary figure 2 Regulation of S100A9 mRNA expression by HBx-mediated NF-κB activation. (a) Real-time PCR analysis for mRNA levels of S100A9 in HepG2 cells transfected with pcDNA3.1-HBx followed by treatment with NF-κB inhibitor BAY 11-7082 (5 μM) for 24 h. (b) Real-time PCR analysis for mRNA levels of S100A9 in HepG2 cells transfected with pcDNA3.1-HBx followed by treatment with siNF-κB p65 for 24 h. ***p<0.001.
